# Predicting Quantitative Genetic Interactions by Means of Sequential Matrix Approximation

**DOI:** 10.1371/journal.pone.0003284

**Published:** 2008-09-26

**Authors:** Aki P. Järvinen, Jukka Hiissa, Laura L. Elo, Tero Aittokallio

**Affiliations:** 1 Biomathematics Research Group, Department of Mathematics, University of Turku, Turku, Finland; 2 Data Mining Research Group, Turku Centre for Biotechnology, Turku, Finland; Michigan State University, United States of America

## Abstract

Despite the emerging experimental techniques for perturbing multiple genes and measuring their quantitative phenotypic effects, genetic interactions have remained extremely difficult to predict on a large scale. Using a recent high-resolution screen of genetic interactions in yeast as a case study, we investigated whether the extraction of pertinent information encoded in the quantitative phenotypic measurements could be improved by computational means. By taking advantage of the observation that most gene pairs in the genetic interaction screens have no significant interactions with each other, we developed a sequential approximation procedure which ranks the mutation pairs in order of evidence for a genetic interaction. The sequential approximations can efficiently remove background variation in the double-mutation screens and give increasingly accurate estimates of the single-mutant fitness measurements. Interestingly, these estimates not only provide predictions for genetic interactions which are consistent with those obtained using the measured fitness, but they can even significantly improve the accuracy with which one can distinguish functionally-related gene pairs from the non-interacting pairs. The computational approach, in general, enables an efficient exploration and classification of genetic interactions in other studies and systems as well.

## Introduction

The systematic mapping of genetic interactions in biological systems has the potential to provide a better understanding of how genes function as networks to carry out and regulate cellular processes. In particular, recent advances in the experimental technologies which allow for the large-scale screening of the effects of combinatorial gene deletions are providing an exciting glimpse into the organization of complex genetic networks in terms of revealing novel interacting cellular components and compensatory pathways involved in many cell functions. Comprehensive maps of genetic interactions in model organisms, such as yeast, may also provide a valuable template for understanding the basic principles underlying the relationships between genotype and phenotype in other populations [1]. In humans, genetic interactions are involved in many complex phenotypes and they contribute to most genetic disorders, but the organization of the underlying networks is largely unknown [2], [3]. Due to their combinatorial nature, the mapping of genetic interactions is highly labor-intensive even in genetically amenable organisms. Efficient computational frameworks are therefore required to underpin the full potential of these experiments.

Several large-scale studies, especially in yeast *Saccharomyces cerevisiae*, have already identified a number of synthetic lethal interactions, in which a combination of two individually non-lethal mutations results in lethality [1], [4]. Genome-wide screening strategies for synthetic sick or lethal interactions, such as those based on synthetic genetic arrays (SGA) or the diploid synthetic lethality analysis by microarray (dSLAM), have successfully been used for providing insights into the nature of genetic robustness [5], and for identifying functional relationships among the genes and pathways [6]. In addition to this rather limited spectrum of observed phenotypes (synthetic sick/lethal *vs.* non-interacting pairs), quantitative phenotypes, such as the relative growth rate of yeast colonies, have recently been explored systematically using high-throughput screening approaches like epistatic miniarray profiling (E-MAP) and genetic interaction mapping (GIM) [7], [8]. The importance of measuring a broader spectrum of genetic interactions when identifying functionally-related genes and pathway organizations has been demonstrated in theoretical and experimental studies [9]–[11]. To provide reliable information on genetic interactions, customized data handling and pre-processing pipelines have been developed for the different screening approaches [12]–[13].

Regardless of the experimental technology used, the screening strategies aim to quantify the extent to which a mutation in one gene modulates the phenotype (or fitness) associated with altering the second gene, either by explicitly measuring or analytically comparing the observed fitness of double-mutants to those of single-mutants. More formally, a genetic interaction between mutants *i* and *j* can be defined by the deviation (*ε_ij_*) of an observed double-mutant phenotype (*P_ij_*) from the expected neutral phenotype of an organism's fitness (*E_ij_*) under the hypothesis that it carries two non-interacting mutations (the null hypothesis). If the fitness *P_ij_* is evaluated in terms of the growth rate of double-mutant *w_ij_*, relative to the wild-type growth rate, and *E_ij_* is a function *g*(*w_i_*, *w_j_*) of the relative single-mutant fitness values *w_i_* and *w_j_*, this definition can be formulated as:
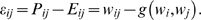
(1)When testing the null hypothesis, a large absolute deviation |*ε_ij_*| provides evidence for genetic interaction, while deviations close to zero indicate non-interacting gene pairs. Significant genetic interactions can further be classified into so-called synergistic interactions (*ε_ij_*<0) and alleviating interactions (*ε_ij_*>0). Synergistic interactions occur when a double-mutant has a more extreme effect on the fitness than would be expected from independent single mutants alone, and can therefore identify e.g. complementary pathways, with synthetic lethality being the extreme case (*w_ij_* = 0). Alleviating interactions, in which the double-mutant phenotype is less severe than expected, can occur, for example, when the first mutation already impairs the function of a whole pathway and thereby masks the effect of the second mutation in the same pathway.

Recently, Mani et al. [14] demonstrated that the product function *g*(*w_i_*, *w_j_*) = *w_i_w_j_* provides a convenient null model in the sense that it yields a distribution with location close to zero and low dispersion over all of the measured deviations. The comparison was based on the principle that, as the vast majority of gene pairs should be non-interacting, the rare gene pairs sharing a specific function should be distinguishable from this background distribution as outlying cases with extreme absolute deviations. Accordingly, it was shown that the observed deviations based on the multiplicative null model were indeed most accurate at identifying functional relationships between the genes [14]. In the present study, we asked two follow-up questions: (1) whether the observed deviations could be estimated directly from the double-mutant phenotypes under the multiplicative model; and (2) whether the prediction of specific functional links could be made at a similar accuracy without utilizing the measurements of single-mutant phenotypes. Under the assumptions that significant genetic interactions are rare and that the multiplicative model is a reasonable approximation in the case of no interaction, we developed a sequential approach that enables a multi-resolution approximation of the double-mutant fitness matrix to address these particular questions, and more generally, to provide a computational framework for exploring genetic interaction datasets.

## Results

### Estimating single-mutant fitness values

As an initial study objective, we sought to assess the accuracy to which the single-mutant fitness vector 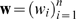 could be estimated directly from the double-mutant fitness matrix 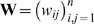. In the quantitative genetic interaction dataset of St Onge et al. [10], which was used in the following results, **w** is a 26-dimensional column vector and **W** is a 26×26-dimensional symmetric matrix. Under the multiplicative null model, Eq. 1 takes the form:
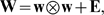
(2)where 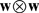 is the tensor product (or outer-product) of the vector **w** with itself, and **E** is the *n*×*n*-matrix comprising the *ε_ij_* values as its elements for each gene pair *i*, *j* = 1,2,…,*n*. In the ideal case, when there are no measurement inaccuracies, the approximation problem of Eq. 2 could easily be solved using the well-established machinery of linear algebra. More precisely, using the spectral decomposition theorem, one can represent any symmetric real matrix as 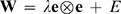, where *λ* is the largest eigenvalue of **W** and **e** is the corresponding eigenvector [15]. Under the unrealistic assumption that there are no genetic interactions among any of the gene pairs, the approximation would in fact be exact, that is, the residual error **E** equals zero. However, as the genetic interaction screens are bound to present with experimental variation, missing data, and hopefully also with significant genetic interactions, the estimation problem must in practice be solved by numerical means.

**Figure 1:**
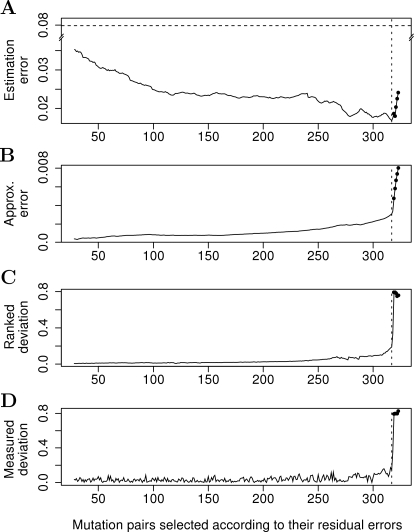
Dynamic behavior of the sequential procedure as a function of mutation pairs. (A) The mean absolute error between the measured single-mutant fitness vector and its estimate when using the selected mutation pairs. The dotted horizontal line indicates the estimation error obtained when using the median over the rows/columns of the double-mutant fitness matrix (average absolute error equals 0.0797). In case significant genetic interactions are rare, median of the colony sizes over all of the double mutants arising from the same single deletion strain provides an estimate of the effect of the particular single-mutant on the growth rate [12]. (B) The approximation error when using the selected mutations pairs to approximate the double-mutant fitness matrix (see Eq. 3 in Methods). The horizontal dotted line indicates the point of steepest increase in the approximation error, *k* = 317, which also gives on average the most accurate estimates of the single-mutant fitness values (average absolute error equals 0.0168). In each panel, the five spots after that line identify the synthetic lethal mutation pairs with double-mutant fitness value of zero. (C) The ranked deviations 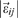 of the mutation pairs (*i*, *j*) defined according to their residual errors (see Eq. 4 in methods). (D) The measured deviations |*ε_ij_*| of the selected mutation pairs obtained using the actual measurements of the single-mutant growth effects.

In the present work, we developed a sequential matrix approximation procedure, which uses increasingly larger subsets of mutation pairs in **W** to provide a series of estimates for **w** as solutions of the weighted least squares problem [16]. During the first rounds, the procedure solves the approximation problem of Eq. 2 using only those mutation pairs that best fit the multiplicative model, and then gradually extends the subset to also include pairs with larger residual errors (see Methods for details). Already when using all but the diagonal and missing entries of the double-mutant fitness matrix in the dataset of St Onge et al. [10], we obtained an estimate relatively close to the actual measured single-mutant fitness vector, as compared to the conventional median estimate (Figure 1A). The estimation accuracy could be markedly improved by excluding those pairs with the largest residual errors from the approximation subset. The pairs having the greatest impact on the approximation error, in fact, corresponded to the five confirmed synthetic lethal pairs (Figure 1B). When the sequential procedure omitted those pairs from the approximation process, an accurate estimate was obtained for each of the single-mutant fitness measurements (see Figure S1). As will be seen in the following subsections, however, the subset of mutation pairs which gives the most accurate estimates of the single-mutant fitness values does not necessarily lead to the best predictive power when identifying functionally related genes.

### Predicting specific functional relationships

**Figure 2:**
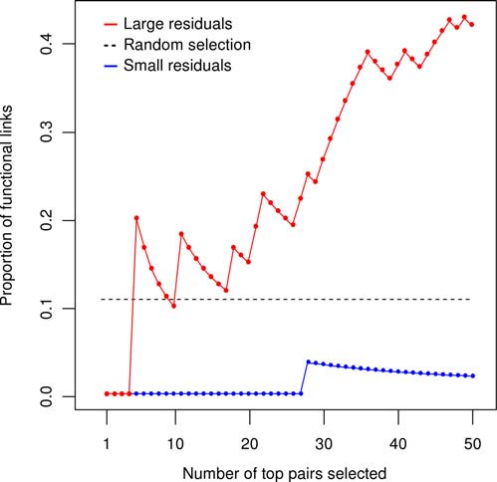
Relationship between the ranking of the mutation pairs and their functional links. The proportion of shared specific functional links among the top mutation pairs, when these pairs were selected in the increasing (red) or decreasing (blue) order according to their residual errors during the approximation process. The dotted line indicates the expected rate of the functional links when selecting mutation pairs at random (the expected proportion equals 0.108).

Beyond the dynamic variability in the estimates of the single-mutant fitness values, the behavior of the approximation procedure with different subsets of mutation pairs revealed another interesting observation: the order in which the mutation pairs were added into the approximation process reflects on average the relative order of their actual measured deviations, even if the measurements of single-mutant fitness were not employed (Figures 1C and D). In fact, the measured deviations *ε_ij_* and the ranked deviations 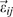 obtained from the approximation procedure were highly correlated (Pearson correlation equals 0.964, see Figure S2). This led us to investigate whether such procedure-ranked deviations could be used instead of the measured deviations when predicting functional links between the mutations. To this end, we took the same set of gene pairs which were found to have a highly specific shared function in the previous study by Mani et al. [14] (see Methods for their definition). Interestingly, the majority of the mutation pairs selected towards the end of the approximation procedure shared a specific function (Figure 2). The rate of the functional enrichment observed among the 50 pairs with the largest 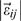 values was significantly higher than expected (*p*<10^−11^), whereas the functional enrichment was exceptionally low among the 50 pairs with the lowest 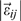 values (*p* = 0.998). These results show that the sequential matrix approximation procedure gives as its by-product a ranking of the mutation pairs that is in good agreement with their likelihood of sharing a specific function.

**Figure 3:**
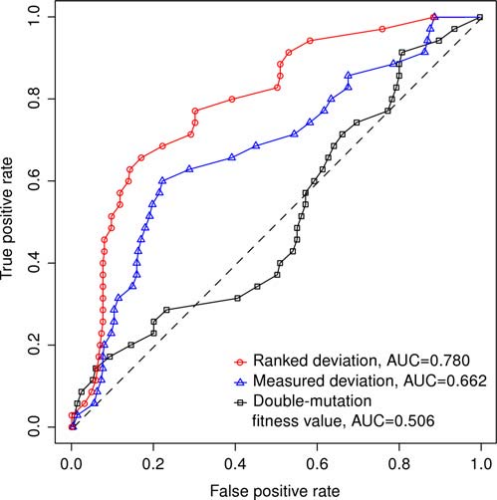
Predicting shared functional links using different measures of genetic interactions. The accuracy of the prediction is evaluated using the receiver operating characteristic (ROC) curves for each measure: ranked deviations (red), measured deviations (blue), and the double-mutant fitness values (black). The true positive rate (TPR, or sensitivity) is the fraction of gene pairs correctly predicted to have functional links, and the false positive rate (FPR, or 1 - specificity) is the fraction of non-functionally linked gene pairs incorrectly predicted to have functional links. The overall prediction performance is summarized using the area under the ROC curve (AUC). For an ideal classifier, TPR = 1, FPR = 0 and AUC = 1, whereas a random classifier has on average AUC = 0.5 (the dotted diagonal line).

To test more systematically whether the prediction of the functional relationships could be made to an accuracy similar to that obtained when using the measured single-mutant fitness values, we assessed how well the ranking based on 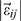 values can discriminate between gene pairs with or without a specific functional link. Similarly as in the original study of St Onge et al. [10], the prediction capability was evaluated using the receiver operating characteristic (ROC) curves that show the relative trade-off between the sensitivity and specificity of the predictions at multiple decision thresholds (Figure 3). Surprisingly, the ranked deviations gave even a better prediction accuracy than the measured deviations according to the area under the ROC curve (AUC) values (AUC = 0.780 vs. AUC = 0.662, *p* = 0.003). The prediction capability was improved systematically at each specificity-level, demonstrating that the procedure could distinguish with high accuracy the functionally related gene pairs over the whole spectrum of the exceptional deviations (Figure 3). The order in which the mutation pairs were included into the approximation process further improved the relative classification power (AUC = 0.789, *p* = 0.002). The prediction accuracy of the double-mutant fitness values alone was similar to that of a random classifier (AUC = 0.506), demonstrating that the normalization by the measured or estimated single-mutant fitness values can, in any case, improve the prediction of the functional links. The estimates achieved using different subsets of mutation pairs can also lead to different degrees of prediction accuracy (Table 1).

**Table 1:** Distribution characteristics for the measured and estimated deviations.

Subset size, *k*	Median deviation	Trimmed mean	Median absolute deviation	Interquartile range	AUC value
Measured	−0.0141	−0.0152	0.0267	0.0169	0.662
28 (Initial set)	0.0000217	0.00107	0.0209	0.0147	0.772
**136**	**0.000687**	**0.000388**	**0.0202**	**0.00958**	**0.810**
208	0.00134	0.00118	0.0141	0.0108	0.773
317	−0.000634	−0.000816	0.0173	0.0120	0.679
323 (All pairs)	−0.000634	0.00142	0.0236	0.0169	0.730

**Figure 4:**
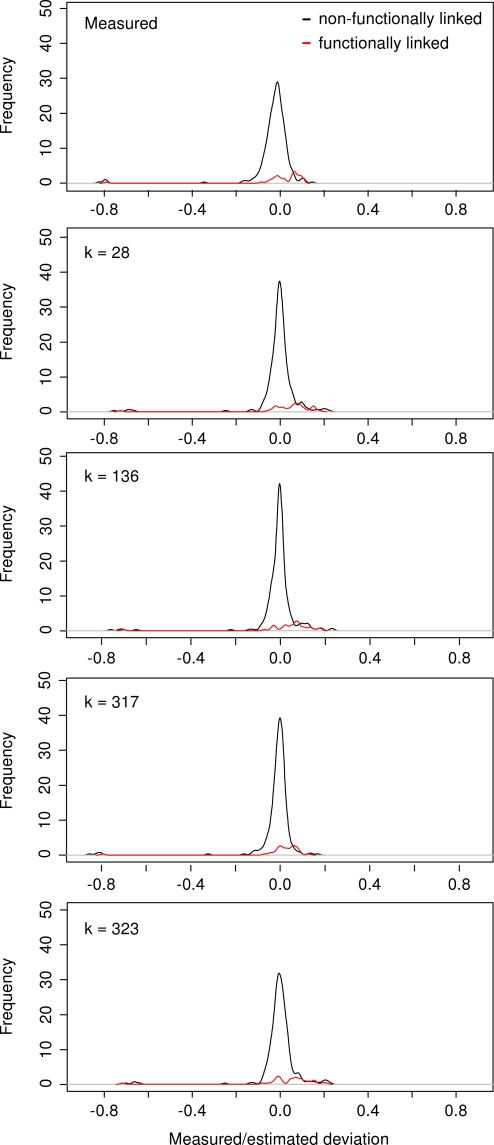
Distributions of the measured and estimated deviations over all mutation pairs. Two distinct distributions are shown for the mutation pairs with specific functional links (red) and for the background pairs not sharing specific functional links (black). The measured deviations *ε_ij_* generate bimodal distribution for the pairs with specific functional links (the upper panel). The estimated deviations 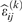 generate background distributions with sharper peaks depending upon the size *k* of the subsets of mutations used in the approximation (the lower panels): *k* = 28 (initial subset of mutations), *k* = 136 (good prediction capability), *k* = 317 (smallest estimation error), and *k* = 323 (all mutation pairs). The five smallest deviation values in each distribution plot correspond to the five synthetic lethal mutation pairs. Table 1 lists the shape parameters of these distributions calculated over all of the mutation pairs. The two distributions in each individual plot are scaled according to their total number of pairs. The non-scaled versions of the same distributions are provided as Figure S3, which allows for better visual comparison between the functionally-linked and the functionally non-linked pairs.

The rows correspond to distributions of the estimated deviations 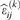 with different sizes of subsets of those gene pairs used in the approximation process (see Figure 4). The distribution of the measured deviations *ε_ij_* is used as a references value for the different parameters (the first row). As robust measures of location (bias) and dispersion (variability), we calculated the trimmed mean and interquartile range, respectively, in addition to the median and median absolute deviation that were used in the previous comparative study [14]. The bold type indicates the subset size which provided the most accurate prediction of the functional links in terms of the area under the receiver operating characteristic curve (AUC).

### Distribution of the estimated deviations

Finally, we investigated how the estimated deviations 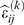 obtained from the approximation procedure at different subset sizes, *k*, are distributed relative to the true deviations *ε_ij_* obtained when using the measured single-mutant fitness values. When comparing the different distributions, we used the same interpretation rule as in the earlier comparison by Mani et al. [14]: an ideal definition of genetic interaction should result in a tight distribution (indicating low variability) that is centered at zero (indicating low bias) for the bulk of the measured interactions (reflecting the background distribution of non-interacting genes). The subset of mutation pairs being used in the approximation process had a marked effect on the distribution of the estimated deviations (Figure 4). Moderate subset sizes generated distributions with a lower bias and variability than those obtained using smaller subset sizes or all of the mutations (Table 1). Surprisingly, the cut-off point, *k* = 317, which gave the most accurate estimates for the single-mutant fitness values resulted in a relatively weak prediction accuracy for the functional links (AUC = 0.679). Although the different measures of location and dispersion gave relatively similar results, the trimmed mean and interquartile range identified the distribution with least background bias and variability at *k* = 136 (Figure 4). The estimated deviations obtained using this particular subset of mutation pairs were also most successful in predicting the functional relationships (AUC = 0.810). These results indicate that the distribution characteristics of the estimated deviations could serve as a guide to choosing the optimal subset of gene pairs for defining genetic interactions in a given dataset. Both the optimal subset size and the overall performance of the method is likely to depend on the properties of the dataset being analysed, including the number of gene pairs and and the degree of their functional homogeneity.

**Figure 5:**
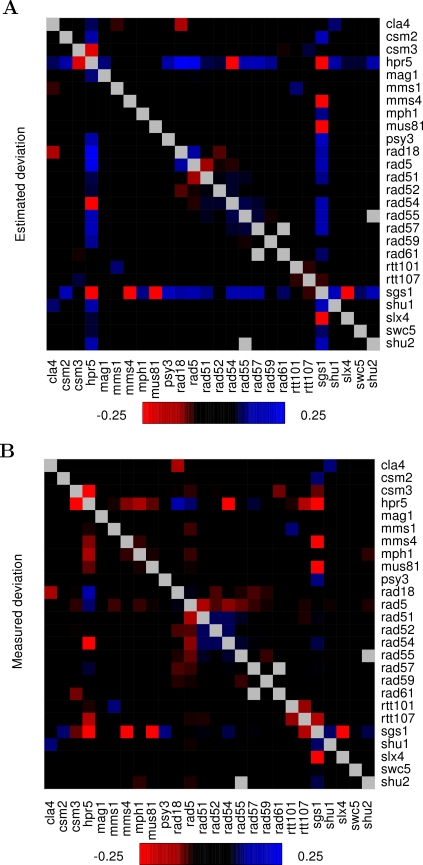
Estimated pairwise deviations *vs.* the actual measured deviations. The color-coded heat map shows the estimated deviations (A) and the measured deviations (B) on a 26×26 grid. The estimated deviations were obtained at the cut-off point 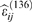, which generates the most ideal distribution and provides the best discrimination between the functionally-linked and the non-functionally linked pairs (see Table 1). While the five confirmed synthetic lethal pairs (sgs1Δ mus81Δ, sgs1Δmms4Δ, sgs1Δslx4Δ, sgs1Δhpr5Δ and rad54Δhpr5Δ) are clearly visible in both of the maps, there are marked differences in the more subtle interaction scores at many places of the matrix between the estimated deviations and the measured deviations *ε_ij_*. Red color corresponds to synergistic interaction scores and blue to alleviating interactions. The grey boxes indicate missing data points.

To investigate how the differences in the distributions are visible in the conventional heat map visualizations, we displayed the color-coded deviations on a two-dimensional grid spanned by the individual mutations. Here, a special emphasis was placed on analyzing the estimated deviations 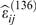, which provided the most ideal definition of genetic interactions in terms of both distribution characteristics and predictive power, relative to the measured deviations *ε_ij_* (Figure 5). In general, the interaction patterns were relatively similar between the measured and estimated deviations. However, the transformation of the double-mutation fitness measurements through the approximation process seemed to emphasize the mutation pairs with exceptionally large absolute deviations (putative genetic interactions), and pointed out especially those pairs having positive deviations (alleviating interactions), while it diminished certain subsets of mutation pairs with negative deviations (synergistic interactions). For instance, a considerable number of double-deletion strains involving either hpr5 or sgs1 mutations showed marked evidence for alleviating interactions in the color map of the estimated deviations (Figure 5A), while these pairs were not identifiable from the original map (Figure 5B). At the same time, the approximation algorithm blotted out certain moderate degree synergistic interactions in the hpr5 deletion strain, including the effects of additional mutations of mms4, mph1, or mus81. Although these and other changes contributed positively to the prediction of functional relationships in the dataset of St Onge et al., further evaluation how well these findings can be generalized beyond this relatively small set of functionally related genes is required on independent datasets.

## Discussion

The growing availability of large-scale genetic interaction datasets is enabling computational methods to systematically explore how genes interact to produce phenotypes on a global network-level. While these datasets yield an unprecedented insight into the organization and function of complex genetic networks, their analysis also poses many challenging computational problems. Using a high-resolution screen of genetic interactions in yeast as an example dataset of St Onge et al. [10], we have demonstrated that the computational approach based on sequential matrix approximations facilitates extraction of pertinent information from the background variation. A key finding of the present work is that the double-mutant fitness matrix alone carries enough information for accurate estimation of the single-mutant fitness values and for prediction of functional relationships among the genes. This makes it possible to avoid performing the single-mutant growth experiments, without compromising - if not even improving – the functional prediction power encoded in the double-mutant measurements. Surprisingly, the subset of mutation pairs which gave the most accurate estimates for the single-mutant fitness values did not lead to the most accurate predictions of the functional links. This may be due to experimental variability, such as differences in growth or screening conditions when measuring the strains carrying either single or double mutations, which may be beyond the capacity of the standard data pre-processing but can be normalized by the sequential approximation procedure. Other possible explanations for this surprising observation include biases in the definition of the gene pairs sharing a specific function or in the targeted set of genes pairs chosen for the particular interaction screen. Further study is therefore needed to confirm whether similar results can be obtained also in larger genetic interaction datasets, in which genes with much wider variety of functional categories are studied.

### Limitations and extensions of the procedure

Perhaps the biggest technical limitation of the present work concerns the heuristic way in which the subsets of mutation pairs were selected for the approximation of the double-mutant fitness matrix. The greedy subset selection scheme was motivated by a similar approach successfully being used in many feature selection problems [17]. An adaptive version of such a forward floating selection method was applied here because of its low computational complexity and because it was capable of excluding the most prominent outliers during the sequential approximation process (Fig. 1). Similarly, despite the weighted least squares matrix approximation algorithm being based on a rather straightforward decomposition method, it was able to reduce some degree of background variation in the data (Fig. 4). However, more sophisticated search and approximation schemes based on e.g. genetic algorithms or simulated annealing should lead to even better estimation and prediction results, or at least reduce the computational complexity. Additional modifications to enhance the present framework either in biological and/or computational terms include using deviations from the expected fitness as weights in the least squares approximation and using the sign of the deviations when including or excluding a mutation pair over the course of the sequential approximation process. While the present results were based on the rank-one approximation only, which enabled the partitioning of pairs of genes into two categories (interacting or non-interacting), utilizing the higher order ranks could allow us to classify the quantitative measurements into several categories, for instance, synergistic and alleviating interactions, or even more fine-grained classification of interactions that can occur between genes [18]. This could also help us also to distinguish those biological modules in which the distribution of genetic interactions does not follow the ideal tight and zero-centered distribution that has been used traditionally [9], [14].

### Future applications and research directions

In spite of the above mentioned technical limitations, the present results support the feasibility of the approximation framework for systematic exploration of genetic interaction data, and warrant its applications to larger-scale datasets, such as those generated with the E-MAP or GIM screens, to confirm whether similar findings can be extrapolated to the genetic interactions data derived from high-throughput technologies. Other quantitative phenotypes or experimental techniques for defining or measuring genetic interactions could, in principle, also be used, although certain modifications will be needed to adapt the procedure to the specific characteristics of each genetic interaction screen. In the larger-scale screens, the gene pairs under analysis can be selected more randomly among a wider range of functions, thus increasing the expected proportion of non-interacting pairs. Accordingly, the more interactions there are being measured, the better the assumptions behind the multiplicative model will be justified, provided that significant genetic interactions remain relatively rare. The many missing values typically occurring in the large-scale screens should not pose a major problem for our approach either, due to its sequential nature being able to adapt to those subsets of double-mutant measurements with the best approximation power. Hence, the approximation approach is likely to yield even better results with larger and unbiased datasets. Similarly, even if the assumption of low frequency of significant interactions may become compromised in more targeted studies, such as the one of St Onge et al, this should not have a major effect on the results as the strongest genetic interaction pairs are effectively filtered out in the sequential estimation process. For such smaller-scale and more targeted genetic interaction studies, a further increase in the performance could be obtained by modifying the null model for non-interacting pairs to take into account the multitude of single-mutants affecting the particular double-mutant fitness value. This is one of the modeling challenges which we aim to tackle in the future.

### Integrative analysis together with other data sources

More generally, the computational approach based on the sequential matrix approximation can provide a principled framework for exploring and classifying genetic networks and interactions using a wide spectrum of global data sources, including the localization, mRNA or protein expression, physical interaction and functional annotation of the proteins encoded by the genes [19]. It has previously been demonstrated that physical protein-protein interactions, in particular, provide useful information that is, by and large, complementary to that obtained from the functional genetic interactions [6], [20]. To reveal the modular structure of the underlying networks and functional organization the multitude of pathways reflected in such large-scale data types, various network partitioning methods have been used to detect either densely- or similarly-connected clusters as well as significantly-repeated motifs in the individual or integrated interaction networks [21]–[24]. However, many open questions still remain about the integrative analysis strategy of these datasets and the most meaningful interpretation of their results. For instance, the extent to which the genetic interaction could be explained by the other information sources, such as protein-protein, protein-DNA, metabolic network and protein structure data [25]–[28], and how these should be efficiently employed when scoring genetic interactions using measures such as the pairwise deviations, the S- and COP-scores, or the correlation and congruence between the interaction patterns [9]–[12], [27]–[29]. Finally, the success of any computational approach for constructing genetic interaction networks is likely to be driven by parallel improvements in the experimental technologies, such as enabling measurement of phenotypic effects in response to the mutation of more than two genes in combination.

## Materials and Methods

### Approximating the double-mutant fitness matrix

For a given subset of mutation pairs, we calculated the matrix approximation of Eq. 2 using the decomposition algorithm by De Leeuw [16]. Formally, we solved the following weighted least-squares optimization problem at each step *l* of the procedure
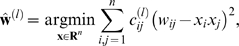
(3)where both the given weight matrix **C** ^(*l*)^ and the double-mutant fitness matrix **W** are symmetric of order *n*. After normalizing for the subset size, the square-root of the objective function obtained as the solution of the optimization of Eq. 3 is referred to as *approximation error* at phase *l*. Using this formulation, the solution **ŵ** ^(*l*)^ minimizes the sum of the squared *residual errors*

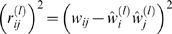
(4)over all of the *k* gene pairs (*i*, *j*) with 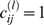 which were involved in the approximation process at the *l* ^th^ step of the procedure. Although the general formulation in Eq. 3 allows for an element of **C** ^(*l*)^ to be any non-negative number, we used binary weights only; in particular, we set the diagonal entries 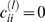 for all *i*, and 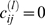 for the missing data points, at each step *l*. Throughout the operation of the approximation procedure, we ensured that the weight matrix remained symmetric.

In the dataset of St Onge et al. [10], there were two missing values because of genetic linkage between the gene pairs involved in the double-deletion strains rad57Δrad61Δ and rad55Δshu2Δ.

### Selecting subsets of mutation pairs for estimation

The subset of mutation pairs used at a particular step of the approximation process was encoded in the binary weight matrix **C** ^(*l*)^, that is, 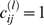 if and only if the mutation pair (*i*, *j*) is used at step *l*. To select increasingly large subsets of non-missing and non-diagonal mutation pairs from the double-mutant fitness matrix **W**, we adapted the floating search method of Pudil et al. [17]. The sequential subset search method is characterized by a dynamically changing number of features included or eliminated at each step. In our implementation, the residual errors were used as the criterion function for adding or deleting mutation pairs. The operation of the forward-type subset selection scheme was organized through the following steps:

1. Set *l*←0 and initialize the weight matrix **C** ^(0)^ (see the next subsection)
2. Estimate **ŵ** ^(*l*)^ using the decomposition algorithm (previous subsection)
3. While there are non-diagonal and non-missing entries with 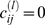

  - select pair (*i*, *j*) with 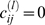 having the smallest residual error 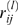 (Eq. 4)
  - set 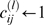, make a new estimate of **ŵ** ^(*l*)^ and re-calculate the residuals 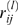

  - if there exists a pair (*i*, *j*) with 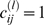 and 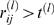, then set 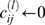

  - set *l*←*l*+1 and repeat step 3

We modified the general subset search method by making the deletion of pairs an adaptive process over the evolution of the subset search. More specifically, the threshold *t* ^(*l*)^ used in the conditional exclusion step 3c is multiplied by 1.5 each time the pair selected for deletion is the same as that added in step 3a. This modification enabled the forward-type algorithm to recover from poor starting tolerance values (we used *t* ^(0)^ = 2×10^−5^ in the present work) or from a poor initialization of the weight matrix (see the next subsection for details), and it also made it possible to increase the size *k* of the subsets up to the maximal size *K*. Figure 1 shows the evolution of the subset selection algorithm from the initial subset configuration to the full set of *K* = 323 mutation pairs in the dataset of St Onge et al. [10].

### Initialization of the weight matrix from the data

The approximation algorithm requires the weight matrix **C** ^(*l*)^ to be non-singular at each stage of the procedure. Therefore, we cannot start the sequential approximation procedure from the empty subset of mutation pairs, but instead must adjust the initialization **C** ^(0)^ = 0 for the dataset under analysis. After calculating the initial residuals 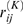 from the whole double-mutant matrix, three types of adjustments were made in the present study: (*i*) for each *i*, we set 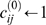 for the pair (*i*, *j*) with the smallest residual 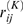 to make all of the rows in **C** ^(0)^ non-zero; (*ii*) we made an additional setting of 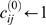 for each identical row pair to reduce the linear dependence among the rows of **C** ^(0)^; and finally (*iii*) we set pairs (*i*, *j*) with 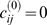 to 1 in the increasing order of their residuals until the determinant of **C** ^(0)^ became non-zero. Every time a pair (*i*, *j*) was added in the initialization steps (*i*)–(*iii*), we also set its transpose entry to one, that is, 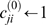, to keep the weight matrix symmetric. In the dataset of St Onge et al. [10], this resulted in an initial weight matrix **C** ^(0)^ with 28 entries of ones.

### Sequential estimation of the pairwise deviations

The *measured deviations ε_ij_* = *w_ij_*−*w_i_w_j_* were estimated in two different ways. The sequential approximation procedure gives as its by-product a surrogate for the deviations in the form of the residual errors of Eq. 4. More specifically, we defined the *ranked deviation* 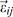 of a mutation pair (*i*, *j*) as its residual error 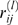 at the last step *l* during which the pair was included into the approximation subset. In this way, even when there are multiple inclusions and deletions of a particular pair during the procedure, we obtained an unambiguous ranking of the pairs according to their 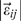 values. This ranking and the corresponding ranked deviations are shown in Figure 1 for all gene pairs of the dataset of St Onge et al. [10], except for the 28 initial mutation pairs.

The second set of estimates was obtained by stopping the sequential approximation procedure at a given subset size, *k*, and by using the estimate of Eq. 3 in place of the measured single-mutant fitness vector **w** in the definition of the deviation in Eq. 1. For a mutation pair (*i*, *j*), this resulted in a sequence of *estimated deviations* 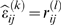 for step *l* at which the size of the approximation subset equals *k*. Note that since the residual errors in Eq. 4 are updated each time that a new pair is added, the estimated deviations can vary considerably as a function of *k*. These estimates are not generally congruent with the ranked deviations. The distributions of the estimated deviations with different subset sizes *k* are illustrated in Figure 4 in the dataset of St Onge et al. [10].

### Quantitative genetic interaction measurements

To evaluate the performance of the sequential matrix approximation procedure in practice, we applied it to a recent high-resolution genetic interaction study of St Onge et al. [10]. This particular study was chosen because it contains quantitative growth-rate measurements of both single- and double-mutant cell populations for a targeted set of 26 genes related to DNA repair in yeast *S. cerevisiae*. The detailed time course fitness measurements were performed in the presence and absence of the DNA-damaging agent methyl methaneusulfonate (MMS). The results of the present study were based on the growth measurements in the absence of MMS. The prediction of functionally related gene pairs was in fact more challenging in this case than in the data measured in the presence of MMS. The measured and estimated single-mutant fitness values and the double-deletion deviations in the dataset are shown in Figure S1 and Figure 5, respectively.

### Defining gene pairs sharing a specific function

Functional links among the 26 genes were defined using the same approach as in many previous genetic interaction studies [6], [10], [14]. Briefly, a term in the Biological Process branch of the Gene Ontology was considered specific if it was associated with fewer than 30 yeast genes, and two genes were considered to have a specific functional relationship if they shared any of those specific terms [14]. This resulted in the set of 35 specific functional links in the dataset of St Onge et al. [10].

### Statistical evaluation of the predictive power

Statistical enrichment of the specific functional links among a set of mutation pairs selected by the sequential approximation procedure was assessed using the standard hypergeometric test. Briefly, if *t* is the number of top mutation pairs selected according to their residual errors, and *M* is the total number of the functionally-related links, then the probability of obtaining at least *m* functionally-related pairs when selecting pairs at random from the set of *K* mutation pairs can be calculated using the cumulative distribution function:
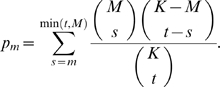



The enrichment for the *M* = 35 functionally-linked pairs among the *t* = 50 mutation pairs selected on the basis of either small (*m* = 1) or large (*m* = 22) residual errors is shown in Figure 2. The dotted line shows the expected rate of the functional links when selecting mutation pairs at random, that is, *M*/*K* = 35/323 = 0.108.

The predictive power of the measured and estimated deviations was assessed using the receiver operating characteristic (ROC) curves that characterize the relative trade-off between the true positive rate (sensitivity) and the false positive rate (1 - specificity). The overall predictive performance was summarized using the area under the ROC curve (AUC). The statistical significance of the difference in the AUC values between two genetic interaction measures was assessed using a custom written algorithm based on the method of DeLong et al. [30]. This nonparametric method uses the theory of generalized *U*-statistics to calculate an estimated covariance matrix and hence it can also take into account the correlated nature of the data. The ROC curves and the corresponding AUC values for the prediction of the 35 functional links in the dataset of St Onge et al. [10] are shown in Figure 3.

## Supporting Information

Figure S1 Estimated vs. measured single-mutant fitness values. The comparison is shown both as histogram and scatter-plot. The two fitness values were highly correlated (Pearson correlation equals 0.952 and the offset and slope of the best fit line are 0.0429 and 0.960, respectively). The estimated values were calculated at the cut-off point k = 317, in which the approximation procedure used all but the diagonal and missing entries of the double-mutant fitness matrix and also omitted those six pairs with the most extreme residual errors (the five synthetic lethal mutations and one plausible synergistic mutation pairs). This point can approximately be identified from the sharp increase in the trace of approximation error (Figure 1B, the vertical dotted line).
(0.01 MB PDF)

Figure S2 Scatter-plot between the measured and ranked deviations. The ranked deviations were highly correlated with the true measured deviations over all of the mutation pairs (Pearson correlation equals 0.964). The inset shows the five synthetic lethal mutation pairs. The dotted diagonal line corresponds to the one-to-one correspondence between the two deviations.
(0.01 MB PDF)

Figure S3 Distributions of the measured and estimated deviations. The non-scaled version of the Figure 4, which can better show the discrimination between the distributions of functionally-linked (red) and functionally non-linked pairs (black).
(0.02 MB PDF)
