# Supplementary figures and images for: Predicting Quantitative Genetic Interactions by Means of Sequential Matrix Approximation
Source: PLoS One. 2008 Sep 26;3(9):e3284. doi: 10.1371/journal.pone.0003284 (PMC2538561; doi:10.1371/journal.pone.0003284)

Single-mutation fitness

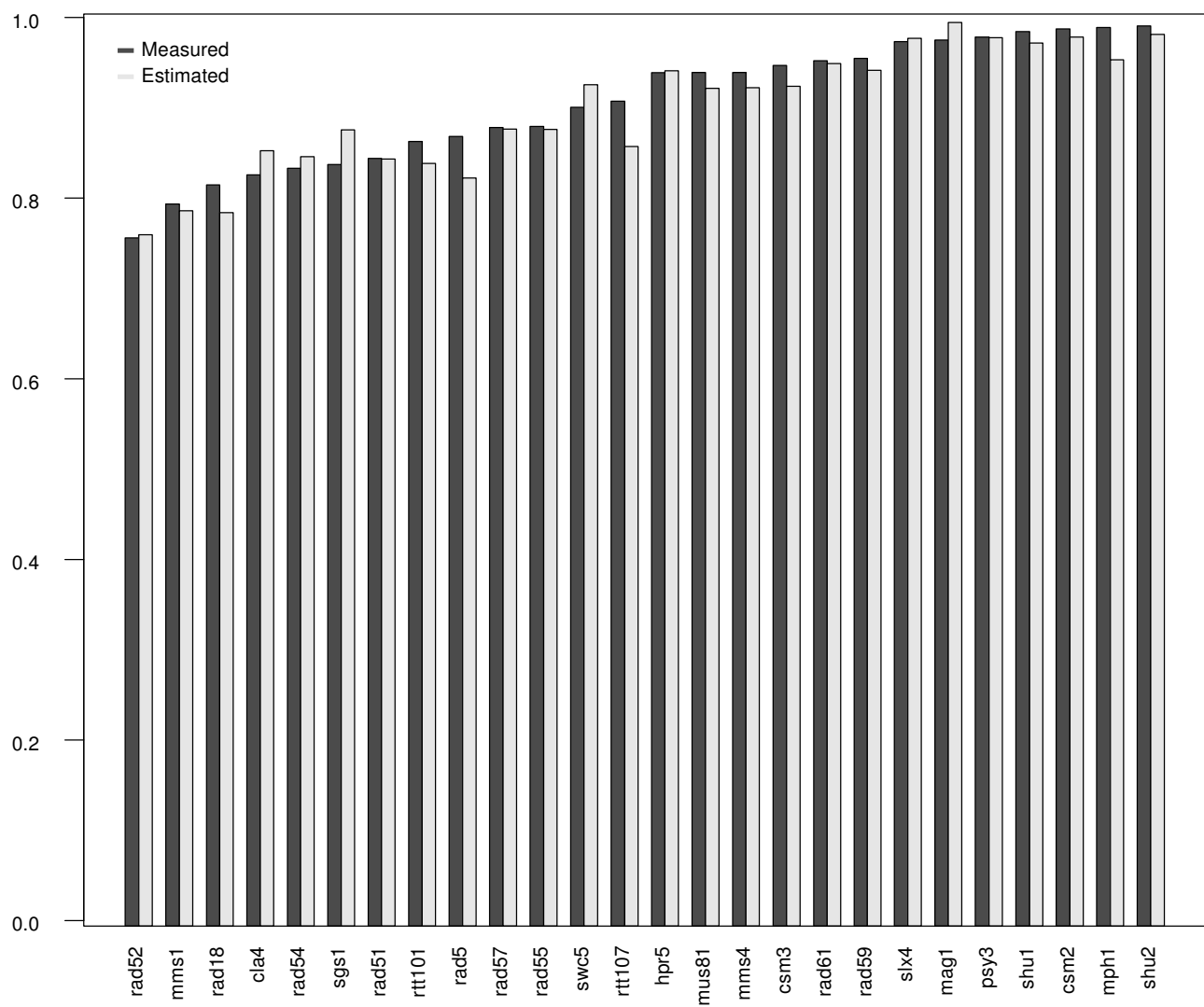

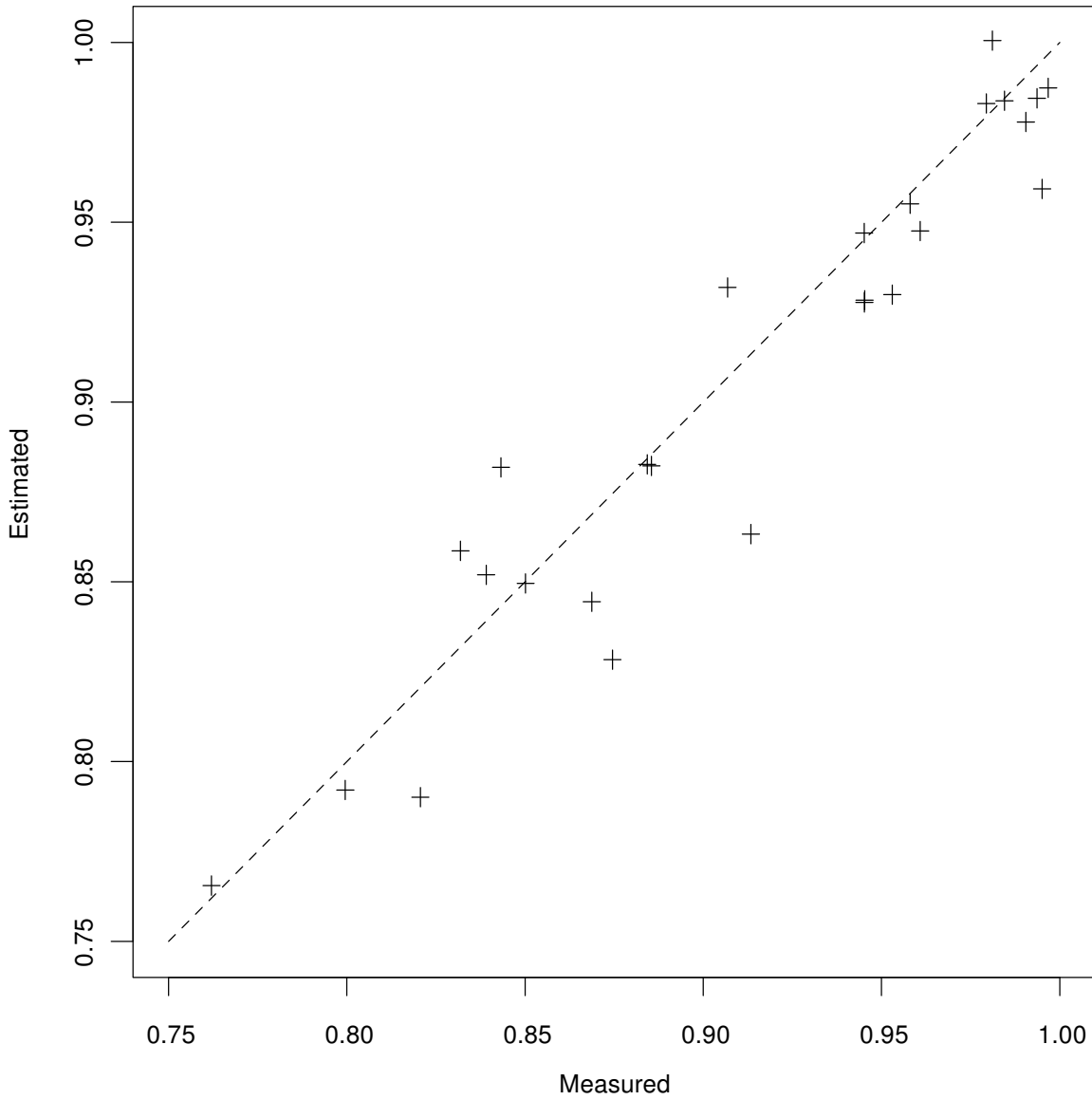

Supplement: Figure S1 — Estimated vs. measured single-mutant fitness values. The comparison is shown both as histogram and scatter-plot. The two fitness values were highly correlated (Pearson correlation equals 0.952 and the offset and slope of the best fit line are 0.0429 and 0.960, respectively). The estimated values were calculated at the cut-off point k = 317, in which the approximation procedure used all but the diagonal and missing entries of the double-mutant fitness matrix and also omitted those six pairs with the most extreme residual errors (the five synthetic lethal mutations and one plausible synergistic mutation pairs). This point can approximately be identified from the sharp increase in the trace of approximation error (Figure 1B, the vertical dotted line). (0.01 MB PDF) [file pone.0003284.s001.pdf]

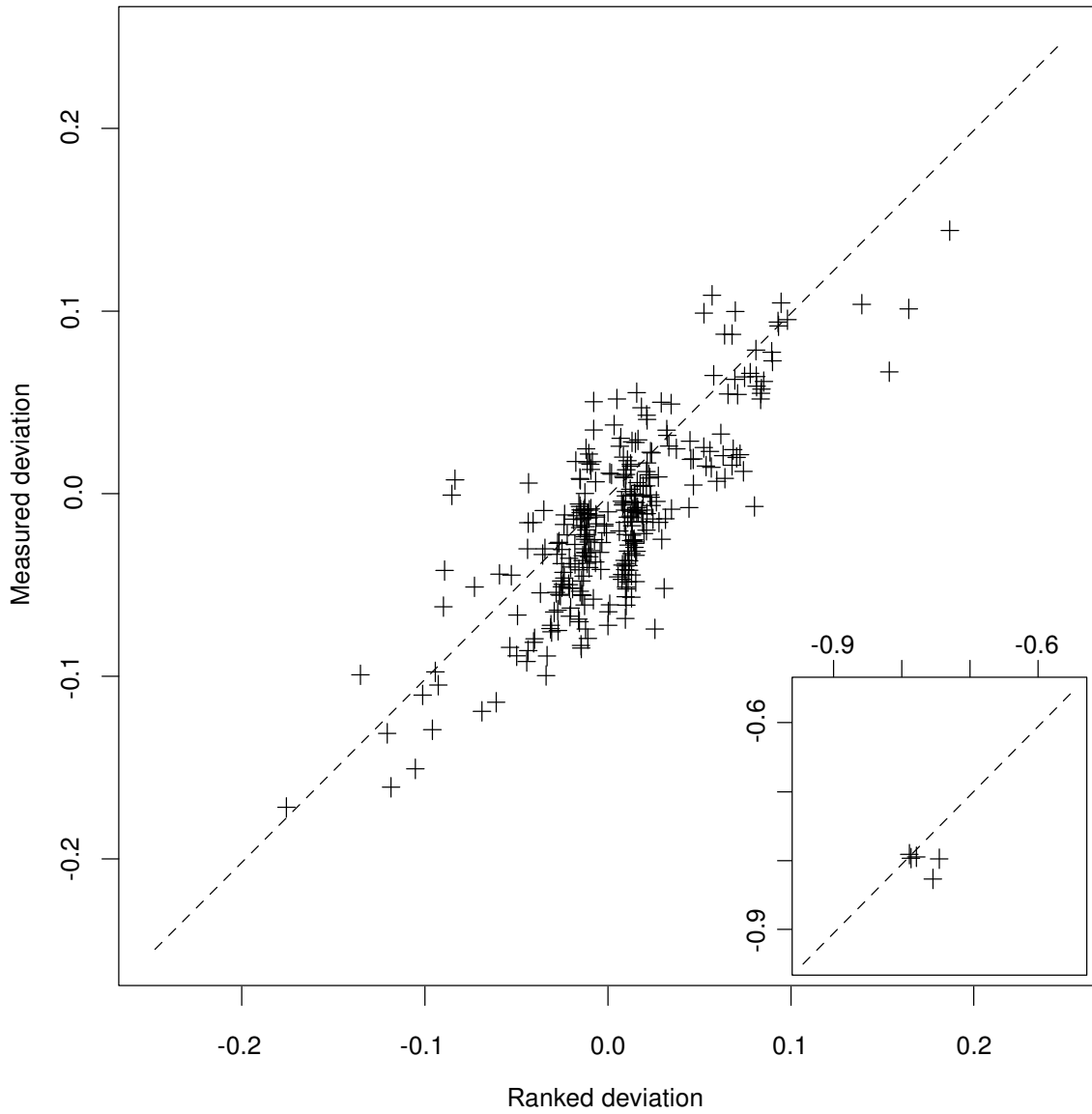

Supplement: Figure S2 — Scatter-plot between the measured and ranked deviations. The ranked deviations were highly correlated with the true measured deviations over all of the mutation pairs (Pearson correlation equals 0.964). The inset shows the five synthetic lethal mutation pairs. The dotted diagonal line corresponds to the one-to-one correspondence between the two deviations. (0.01 MB PDF) [file pone.0003284.s002.pdf]

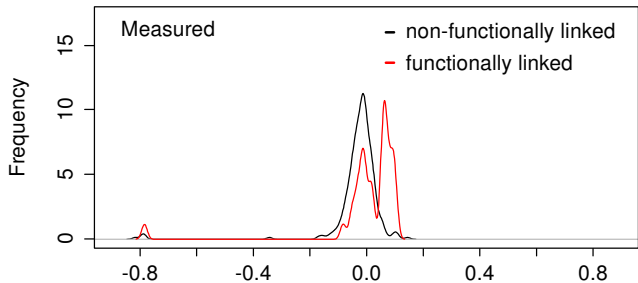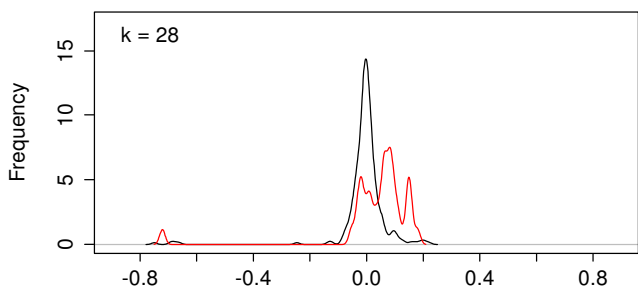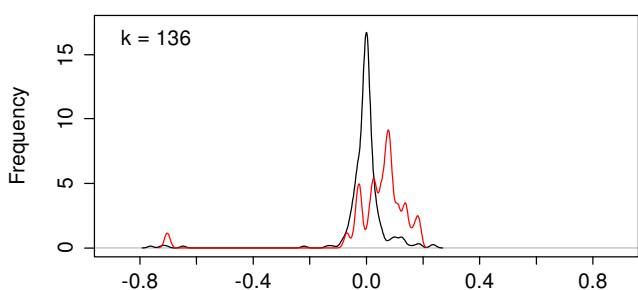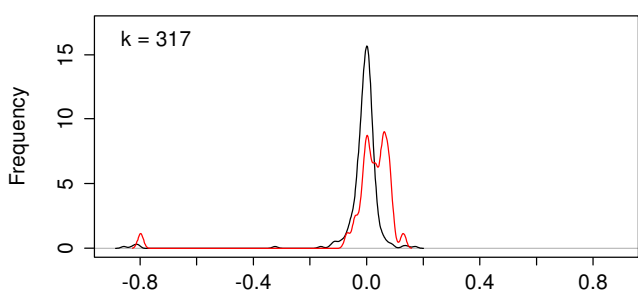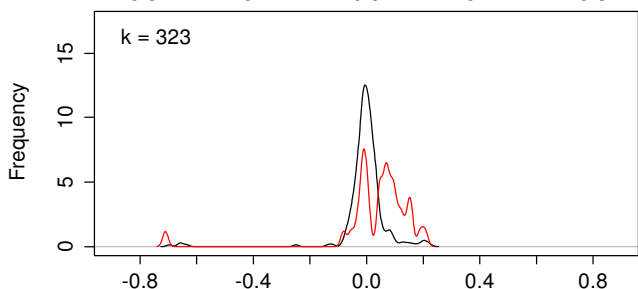

Measured/estimated deviation

Supplement: Figure S3 — Distributions of the measured and estimated deviations. The non-scaled version of the Figure 4, which can better show the discrimination between the distributions of functionally-linked (red) and functionally non-linked pairs (black). (0.02 MB PDF) [file pone.0003284.s003.pdf]
